# Pregnancy during COVID-19: social contact patterns and vaccine coverage of pregnant women from CoMix in 19 European countries

**DOI:** 10.1186/s12884-022-05076-1

**Published:** 2022-10-08

**Authors:** Kerry L. M. Wong, Amy Gimma, Enny S. Paixao, CoMix Europe Working Group, Daniela Paolotti, André Karch, Veronika Jäger, Joaquin Baruch, Tanya Melillo, Henrieta Hudeckova, Magdalena Rosinska, Marta Niedzwiedzka-Stadnik, Krista Fischer, Sigrid Vorobjov, Hanna Sõnajalg, Christian Althaus, Nicola Low, Martina Reichmuth, Kari Auranen, Markku Nurhonen, Goranka Petrović, Zvjezdana Lovric Makaric, Sónia Namorado, Constantino Caetano, Ana João Santos, Gergely Röst, Beatrix Oroszi, Márton Karsai, Mario Fafangel, Petra Klepac, Natalija Kranjec, Cristina Vilaplana, Jordi Casabona, Christel Faes, Philippe Beutels, Niel Hens, Christopher I. Jarvis, W. John Edmunds

**Affiliations:** 1 Department of Infectious Disease Epidemiology, London School of Hygiene and Tropical Medicine, Keppel Street, London, WC1E 7HT UK; 2 Data Science Institute and I-BioStat, Hasselt University, Hasselt, Belgium; 3 Centre for Health Economics Research and Modelling Infectious Diseases, Vaccine and Infectious Disease Institute, University of Antwerp, Antwerp, Belgium

**Keywords:** Pregnancy, COVID-19, Contact survey, Social contact, Lockdowns, Europe

## Abstract

**Background:**

Evidence and advice for pregnant women evolved during the COVID-19 pandemic. We studied social contact behaviour and vaccine uptake in pregnant women between March 2020 and September 2021 in 19 European countries.

**Methods:**

In each country, repeated online survey data were collected from a panel of nationally-representative participants. We calculated the adjusted mean number of contacts reported with an individual-level generalized additive mixed model, modelled using the negative binomial distribution and a log link function. Mean proportion of people in isolation or quarantine, and vaccination coverage by pregnancy status and gender were calculated using a clustered bootstrap.

**Findings:**

We recorded 4,129 observations from 1,041 pregnant women, and 115,359 observations from 29,860 non-pregnant individuals aged 18–49. Pregnant women made slightly fewer contacts (3.6, 95%CI = 3.5–3.7) than non-pregnant women (4.0, 95%CI = 3.9–4.0), driven by fewer work contacts but marginally more contacts in non-essential social settings. Approximately 15–20% pregnant and 5% of non-pregnant individuals reported to be in isolation and quarantine for large parts of the study period.

COVID-19 vaccine coverage was higher in pregnant women than in non-pregnant women between January and April 2021. Since May 2021, vaccination in non-pregnant women began to increase and surpassed that in pregnant women.

**Interpretation:**

Limited social contact to avoid pathogen exposure during the COVID-19 pandemic has been a challenge to many, especially women going through pregnancy. More recognition of maternal social support desire is needed in the ongoing pandemic. As COVID-19 vaccination continues to remain an important pillar of outbreak response, strategies to promote correct information can provide reassurance and facilitate informed pregnancy vaccine decisions in this vulnerable group.

**Supplementary Information:**

The online version contains supplementary material available at 10.1186/s12884-022-05076-1.

## Introduction

The physiological changes and relative immunodeficiency during pregnancy increase vulnerability to outbreaks of emerging infectious diseases for both the mother and foetus. Although being pregnant is not an additional risk for getting COVID-19, if infected, pregnant women are five times more likely to be hospitalized compared to non-pregnant women of a similar age [1]. A review of 192 studies showed that compared to pregnant women without COVID-19, those with the disease had increased odds of intensive care unit (ICU) admission (odds ratios (OR) of 18.6 (95%CI = 7.5–45.8) and maternal death (OR = 7.5, 95%CI = 1.1–7.5) [1]. The odds of preterm birth (OR = 1.5, 95%CI = 1.1–1.9) and admission to neonatal ICU (OR = 4.9, 95%CI = 1.0–12.9) were higher in babies born to mothers with COVID-19 versus those without [1]. Evidence of increased risk of stillbirths with COVID-19 infection has also been reported in large studies in the United States (relative risk = 1.9, 95%CI = 1.7–2.2) and United Kingdom (OR = 2.2, 95%CI = 1.6–3.1) [2, 3].

The World Health Organization (WHO) recommends pregnant women and those around them to take precautions to protect themselves against COVID-19. Both the WHO and the European Centre for Disease Prevention and Control advise pregnant women to avoid crowded and confined indoor places, limit in-person contacts, and ask others to take a COVID-19 test before meeting up [4, 5]. These guidelines, alongside other social distancing and non-pharmaceutical interventions (NPIs) implemented by governments for the public – e.g., restrictions on events and lockdown/”stay-at-home” orders – can reshape social behaviour and reduce individuals’ opportunities for social support and connection, a key buffer for risk of general and psychosocial wellbeing especially during pregnancy [6]. As the pandemic worsened in early spring 2020, pregnant women reported increased levels of stress, fear and uncertainty [7]. However, evidence on how pregnant women altered their contact patterns in response to social distancing guidelines and restrictions has remained scarce.

In addition to social isolation and stress, the development and authorisation of the COVID-19 vaccines that are safe to use in pregnancy are of particular importance. Multiple vaccine trials (excluding pregnant participants) had kicked off soon after the onset of the pandemic and in the months that followed, several large trials announced positive results: that the vaccines triggered immune responses in humans [8]. In Europe, the United Kingdom (UK) was the first country to approve a COVID-19 vaccine for emergency use on 2 December 2020, with their national vaccination campaign starting a few weeks later. Priority groups included elderly people, healthcare workers, and the clinically vulnerable, and excluded women in pregnancy [9]. This exclusion was in part due to limited data, as the best evidence concerning COVID-19 vaccine safety for pregnant women, at the time, were those from animal studies and previous knowledge about vaccination in pregnancy. In January 2020, the priority list extended to include pregnant women who were at increased risk from being severely unwell with COVID-19 (e.g., those with congenital heart conditions) or those at increased risk of infection due to their occupation (e.g., frontline healthcare workers). As more relevant safety data emerged, pregnant women began to receive recommendation for vaccination together with their age cohort in April 2021, and were subsequently put on the priority list in December 2021 [10, 11]. At the individual level, systematic reviews and national studies both revealed mixed perceptions and acceptance of the vaccine among pregnant women [12–14]. Factors affecting acceptance include those related to awareness of vaccine safety, and the way in which safety information is disseminated [14].

The COVID-19 pandemic has severely disrupted access to social support, and left many pregnant women concerned about contracting the virus and getting vaccinated against COVID-19. Data on the effect of the pandemic on pregnant women’s social contact behaviour has remained limited. In this study, we collected data over the course of the COVID-19 pandemic in 19 countries in the European region. We describe the impact of the pandemic, focusing on social contact and social isolation among women reported as being pregnant, their risk perception, and their vaccination coverage over time and across various levels of government restrictions. Our findings offer an important multi-country overview of the experience of this vulnerable group during the COVID-19 pandemic.

## Methods

### Ethics statement

Participation in this opt-in study was voluntary, and all analyses were carried out on anonymised data. In the UK, the study was approved by the ethics committee of the London School of Hygiene and Tropical Medicine (Reference number: 21795). In the other participating countries, any necessary approval from institutional review boards and local ethical committees were obtained, details of which can be found in a previous publication [15].

### Study design and setting

CoMix is an online longitudinal, social contact survey that follows individuals in 19 European countries over the course of the COVID-19 pandemic. The survey asks people aged 18 or above about their awareness, perceptions, social contacts, and health condition over the course of the COVID-19 pandemic. Study participants are invited to the survey and asked to respond every one to two week(s) apart. In each country, a nationally representative sample was recruited by the market research company Ipsos-MORI or a local vendor using quota sampling based on age, gender, and geographic region, and when possible, socioeconomic status to reflect the distribution within the population. Recruitment was conducted through web advertising and email campaigns.

The design of the CoMix survey is based on the POLYMOD contact survey [16] with additional questions about work attendance, household composition, self-isolation and -quarantine due to COVID-19, and COVID-19 vaccination (since December 2020), among others. Details of the CoMix study including the protocol, further methodological details and survey instrument for the UK panels have been published previously [17]. A copy of the questionnaire is available on https://github.com/wongkerry/comix_preg/tree/main/questionnaire.

CoMix was first launched in March 2020 in the UK, Belgium, and the Netherlands [18, 19]. In the UK, two panels of respondents are asked to respond once every two weeks, in alternating weeks. Initially, each panel consisted of about 1500 participants, increasing to about 2500 participants each week from August 2020. We recruited new participants on a rolling basis as existing participants dropped out of the study. Participants were included for a maximum of 7–10 survey rounds. Further details of CoMix in the UK have been published elsewhere [19, 20]. In the other CoMix countries the sample size were smaller. To the original three CoMix countries, initially 7 countries (Austria, Denmark, France, Italy, Poland, Portugal, and Spain) between December 2020 and April 2021, then 6 countries between February 2021 and October 2021 (Finland, Greece, Lithuania, Switzerland, and Slovenia), and lastly 4 countries between May 2021 and October 2021 (Hungary, Slovakia, Estonia, and Croatia) were added [15]. In each of these countries, the initial panel consisted of at least 1500 participants, who were invited to 7 survey rounds. The original CoMix survey was translated into the local languages.

### Study participants

In this analysis, we included pregnant women (self-identified) aged 18–49 years, and non-pregnant women and men of the same age who reported to have no risk factors for serious symptoms if they contracted COVID-19. We used self-reported pregnancy status to identify pregnant women. Women who had reported not being pregnant for all surveys they completed were considered non-pregnant.

### Data

We combined data on social contacts, risk perception, status, mitigation, and COVID-19 vaccination from the participants of the CoMix survey and information on non-pharmaceutical interventions (NPI) in the study countries from the Oxford Coronavirus Government Response Tracer (OxCGRT) project [21], which provides a systematic way to track government responses to COVID-19 across countries.

### Reporting of contacts (CoMiX) and isolation or quarantine due to COVID-19

Participants reported social contacts that occurred on the day prior to the survey. The details of reporting have previously been documented [19]. Briefly, participants reported contacts by listing each individual or as a total number of contacts made by setting. A direct contact is defined as anyone who met the participant in person with whom at least a few words were exchanged in close proximity, or physical contact was made. We grouped reported contacts by the settings in which they were made – at home and outside of the home setting; we further distinguished contacts made outside of the home setting as contacts made at work, contacts made in social settings and contacts made elsewhere. Social settings were taken as someone else’s house, a place of worship, at a shop for non-essential items, at a place of entertainment such as a restaurant, bar, cinema, at a place for sports, and other outside locations such as in a park or in the countryside. We also asked if participants had been in “isolation or quarantine due to coronavirus (Covid-19)?” in the last seven days. Isolation or quarantine could include staying at home after potential exposure to an infected case, on return from a trip abroad, or separation from people who are not infected (including household members), either at home or in a facility.

### Risk perception, status, mitigation and COVID-19 vaccination (CoMiX)

In addition to social contacts, participants were asked to respond to statements regarding their perception of risk. Participants were asked to respond to the statements:

i) “I am likely to catch coronavirus”,
ii) “I am worried that I might spread coronavirus to someone who is vulnerable”, and
iii) “Coronavirus would be a serious illness for me”.

The responses were captured with the likert scale of “Strongly Agree”, “Tend to Agree”, “Neutral”, “Tend to Disagree”, and “Strongly Disagree”. Participants were also asked whether they wore a face covering.

Since December 2020, we added questions on vaccination against COVID-19. Participants were asked “Have you been vaccinated against the virus that causes Coronavirus (COVID-19)?”, or “Have you had any new doses of the vaccination since you last completed the survey?”. From the day of survey response, participants who responded they had received the vaccination in either of these two questions were considered vaccinated (either partially vaccinated or fully vaccinated).

### NPIs (OxCGRT)

We extracted data on NPIs from the OxCGRT project [21] as measured by an overall stringency index (SI) in each of the study counties. SI is calculated using eight containment and closure policies (school closing, workplace closing, cancelling of public events, restrictions on gathering sizes, closing public transport, stay at home requirements, restrictions on internal movement and international travel controls), plus an indicator recording public information campaigns. SI takes values from 0 (least strident restrictions) to 100 and provides a systematic way to quantify the strictness of “lockdown style” policies aimed primarily at restricting behaviours. In April 2020 in the UK, for instance, all four nations were in the lockdown with education establishments, non-essential retail, alongside the leisure and hospitality sectors closed. People were required to only leave their house for essential shopping or medical needs, or in order to undertake one form of exercise per day. SI at the time was 80. In July 2021, on the other hand, SI was approximately 30 when only few restrictions were in place (Figure S1).

## Statistical analysis

R version 4.0.5 was used for all analyses and the code and data are available on https://github.com/wongkerry/comix_preg.

We presented summary statistics of age, household size, employment status, and occupation (available only in some countries) of the study sample by pregnancy status.

We calculated the predicted mean contacts in different settings (all settings, home, outside of home, work, and other social settings) using a generalized additive mixed model (GAMM) [22, 23]. We assumed reported contacts followed a negative binomial distribution, modelled using a log link function, with a random effect for participants by pregnancy status and gender. The predictions were adjusted for employment status (full-time, part-time, and self-employed) and year-month, and weighted by weekday.

The proportion and associated confidence intervals of participants in isolation or quarantine, agreed/strongly agreed to statements related to perception of risk, the use of face-covering, and vaccination coverage over time with 1000 samples using clustered bootstrapping [21]. Each participant was sampled with replacement and then all observations for selected participants were included in bootstrapped samples to account for dependency from repeated observations of the same participants. This calculation was based on a moving window over two-weeks, overlapping intervals to increase the sample size per estimate and to include all participants from simultaneously running panels. We assess vaccination coverage in all countries except for the UK together due to small sample size in each of the other individual countries. The status of vaccination policy for pregnant women between March and September 2021 in each country is given in Figure S2 [24].

## Results

### Participant characteristics

Overall, we recorded surveys completed by 30,901 participants aged 18–49 years between March 2020 to September 2021, 1,041 (3.4%) of whom were pregnant women (Table 1). The mean age was approximately 33.8 years in all groups. Over half (54.8%) of all participants reported to be in full-time employment, with a greater proportion of men reported being employed fulltime (64.1%). The mean household size was 3 people.

**Table 1:** Characteristics of study participants and distribution of completed surveys by country and stringency index of NPIs

	**All participants**	**Pregnant women**	**Non-pregnant women**	**Men**
**Number of participants**	30,901	1,041	17,107	12,753
**Mean age**	33.8	31.2	33.2	34.8
**Employment status**				
Employed full-time	54.8%	56.1%	47.2%	64.1%
Employed part-time	12.3%	16.0%	16.3%	6.6%
Self-employed	5.4%	4.7%	4.6%	6.5%
Student	12.2%	4.0%	13.7%	10.8%
Not employed	15.7%	19.1%	18.2%	12.0%
**Mean household size**	2.9	2.9	2.9	2.8
**Household composition**				
At least one person aged ≤ 4	16.9%	28.9%	16.9%	15.9%
At least one person aged 5–11	25.0%	23.7%	24.7%	25.4%
At least one person aged 12–19	30.8%	17.1%	31.5%	31.0%
At least one person aged 70 +	6.5%	3.4%	5.6%	7.9%
**Occupation**^a^				
Health professionals	5.9%	9.9%	6.5%	2.3%
Teaching professionals	6.1%	9.3%	8.3%	4.1%
**Number of completed surveys**	119,488	4,129	64,148	51,211
**Completed by UK participants**	45.5%	54.8%	48.7%	40.9%
**Completed surveys by Stringency Index (SI)**				
< 50	13.6%	12.2%	12.9%	14.5%
51–60	12.7%	14.3%	13.4%	11.9%
61–70	33.9%	34.0%	33.9%	33.8%
71–80	22.4%	22.0%	22.4%	22.5%
81 +	17.4%	17.5%	17.5%	17.3%

^a^Available in Poland, France, Hungary, Italy, Portugal, Spain, Switzerland, and the United Kingdom

**Fig. 1:**
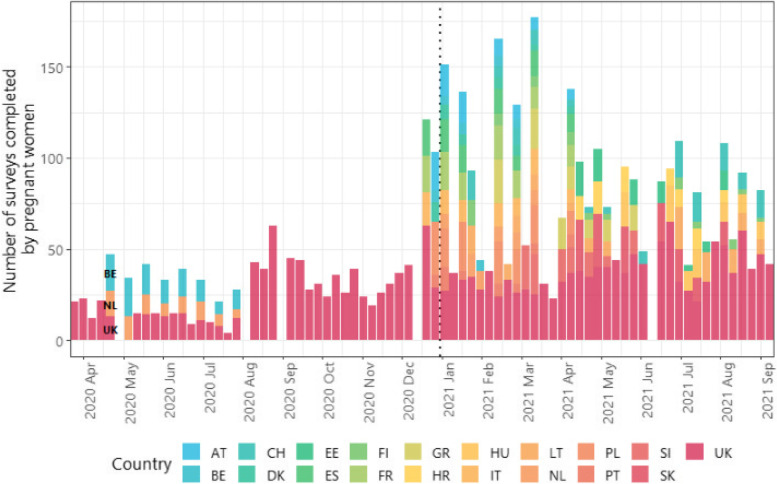
Number of surveys completed by pregnant women over time by country between 23 March 2020 and 12 September 2021. Not all countries are labelled. The dashed line represents 1^st^ January 2021

Over the entire study period, the participants completed 119,488 surveys, 4,129 of which by pregnant women. Nearly half (45.5%) of the surveys were completed by participants in the UK, with each of the other 18 countries contributing between 1.0% to 5.2% (Table S1). Prior to late-December in 2020, the study sample was based in the UK, Belgium, and the Netherlands (Fig. 1). The study sample included more surveys completed by participants from other European countries since late-December 2020 – e.g., Italy, France, Spain, Denmark, Poland, and Austria.

Nearly half (39.8%) of the surveys were completed when NPIs were stringent (Stringency Index (SI) of 71 +), with 13.6% completed when most restrictions were relaxed, and SI was low.

### Mean contacts

The mean number of daily contact reported over the entire study period was slightly lower in pregnant women (3.6, 95%CI = 3.5–3.7) than in non-pregnant women aged 18–49 (4.0, 95%CI = 3.9–4.0), and men of same age (3.7, 95%CI = 3.7–3.8) (Table 2). Contacts at home were approximately 1.5 in all groups. Contacts made at work were lower among pregnant women (0.9, 95%CI = 0.8–1.1) than in non-pregnant individuals (1.3–1.4). Contacts made at social settings and outside of the participants’ household were higher among pregnant women (0.7, 95%CI = 0.6–0.7) than in non-pregnant people (0.5–0.6).

**Table 2:** Bootstrap crude mean contacts with 95% confidence interval of bootstrapping

	**Pregnant women**	**Non-pregnant women** **aged 18–49**	**Men aged 18–49**
**All settings**	3.6 (3.5–3.7)	4.0 (3.9–4.0)	3.7 (3.7–3.8)
**Contacts at home**	1.6 (1.5–1.6)	1.6 (1.6–1.6)	1.5 (1.4–1.5)
**Contacts not at home**	2.1 (1.9–2.2)	2.3 (2.3–2.4)	2.3 (2.2–2.3)
At work	0.9 (0.8–1.1)	1.3 (1.3–1.4)	1.4 (1.3–1.4)
At social settings	0.7 (0.6–0.7)	0.6 (0.6–0.6)	0.5 (0.5–0.5)

### Contacts by setting and NPIs

**Fig. 2:**
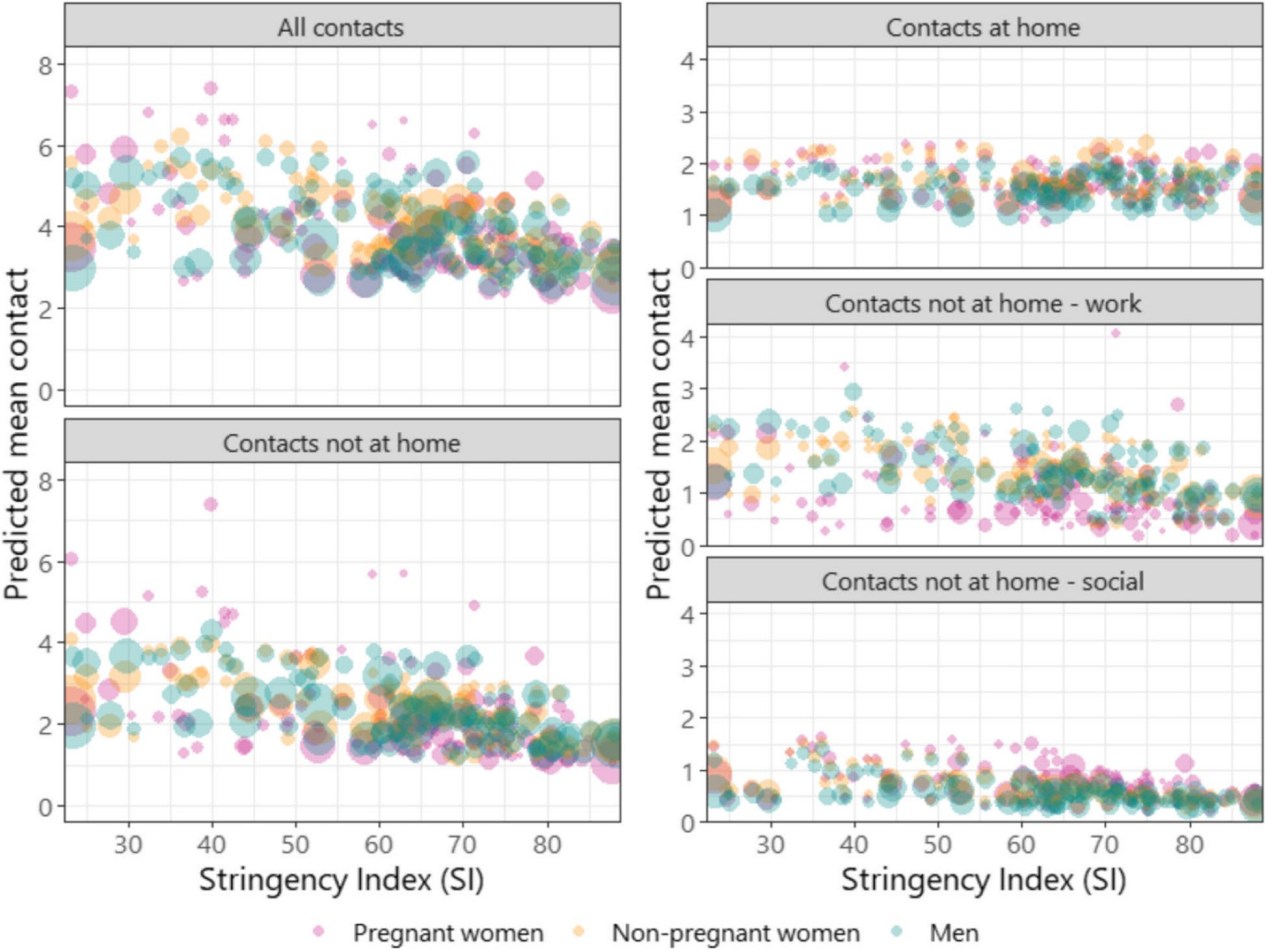
Predicted mean contact in different settings and by different levels of the Stringency Index. Predicted mean contact is adjusted for employment status and month. Every point represents one observation in a country. Size of the bubbles is scaled to the number of completed surveys by pregnancy status and sex

Contacts varied by settings as well as the strictness of the NPIs that were put in place. Home contacts remained consistent at 1.0–2.5 for pregnant women, non-pregnant women and men across all levels of NPI strigency (Fig. 2), contacts not at home declined in all groups. Pregnant women generally made the least contacts outside of the home setting at all levels of NPIs, driven primarily by fewer contacts made at work – in most cases < 2 and at high SI < 1 work contacts vs. 2–3 among non-pregnant people. In social settings, however, pregnant women reported more contacts than non-pregnant people at all levels of restrictions.

**Fig. 3:**
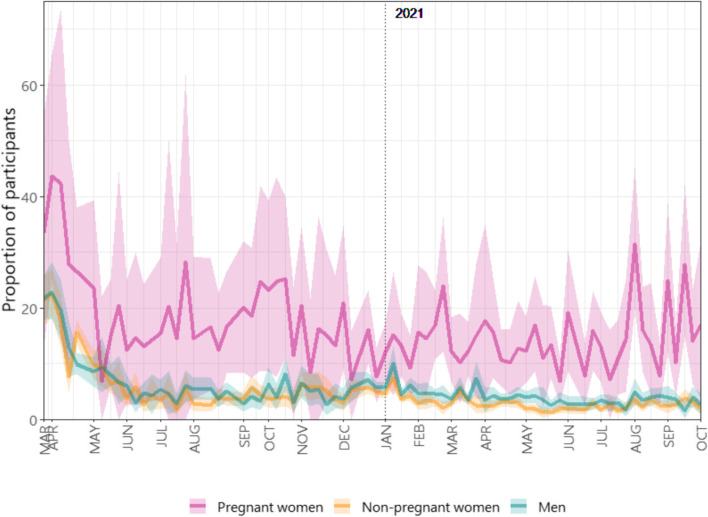
Proportion of participants reporting in isolation or quarantine due to COVID-19 with 95% bootstrap-based confidence interval

Further to making generally fewer contacts, a consistently greater proportion of pregnant women reported being in isolation or quarantine due to COVID-19 – approximately 20%-40% pregnant women versus < 20% in non-pregnant women and men prior to May 2020, and 15% versus 5% since May 2020 (Fig. 3).

### Risk Perception and use of face-covering

**Fig. 4:**
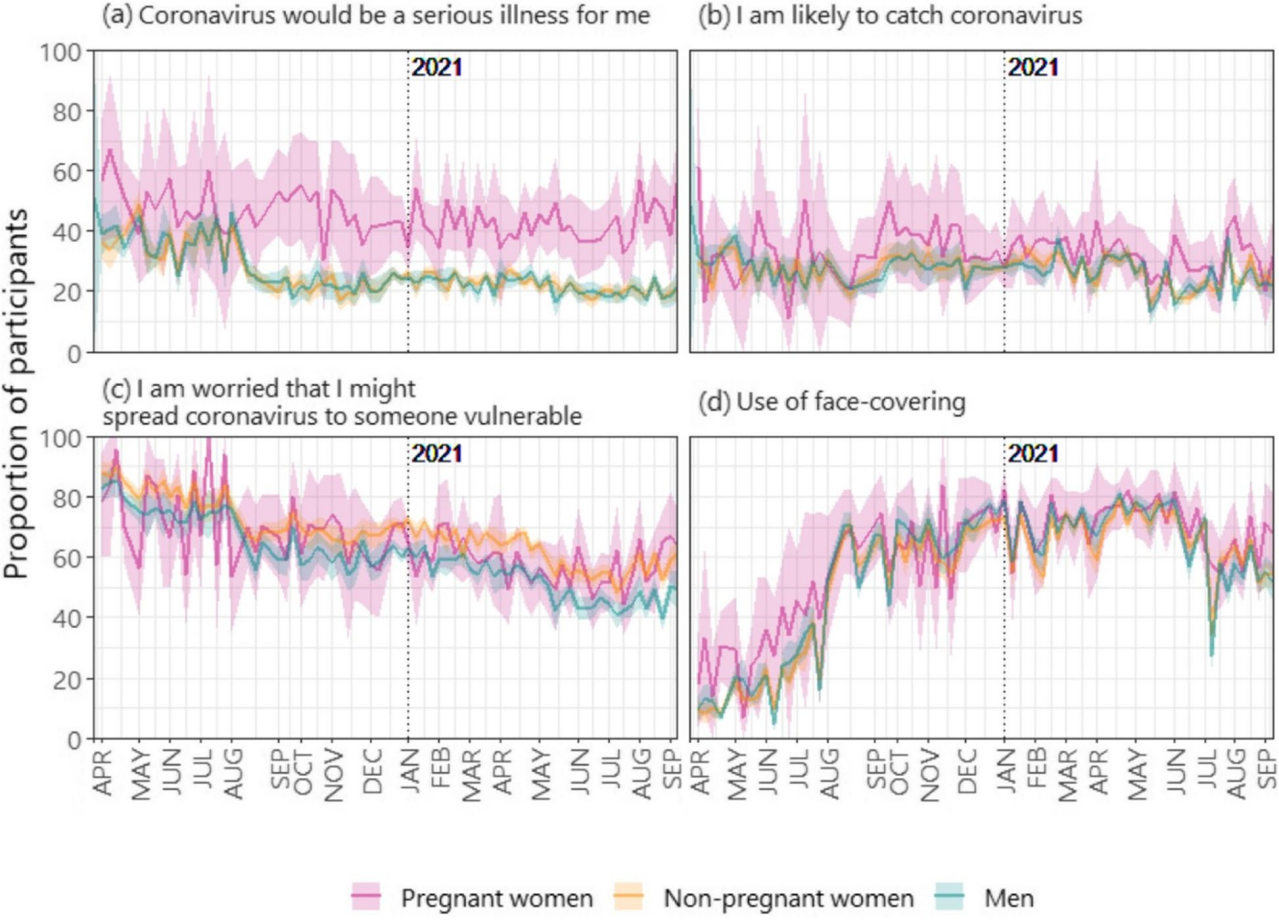
The proportion of participants who answered; with bootstrap-based 95% confidence intervals

Approximately 45–50% of pregnant women answered “Agree” and “Strongly agree” to a statement indicating that coronavirus would be a serious illness for them throughout the entire study period (Fig. 4a). On the other hand, the proportion of non-pregnant people who agreed and strongly agreed with the statement dropped from approximately 35% and maintained at approximately 25% since August 2020.

The proportion who answered “Agree” and “Strongly agree” to the statement “I am likely to catch coronavirus” remained consistent at roughly 25% over time and across both pregnant and non-pregnant individuals (Fig. 4b). The proportion of participants who were worried that they might spread coronavirus to someone vulnerable declined over time in all groups (Fig. 4c). There was some evidence to suggest that slightly more non-pregnant women were more worried that they might spread coronavirus to someone vulnerable (as compared to men).

The use of face covering was 15–20% points higher among pregnant women (30–50%) compared to non-pregnant people between April and August 2020 (Fig. 4d). There was a sharp increase to roughly 70% use of face-covering reported by all groups in August 2020. Usage of face-covering then remained consistent in all groups until a decline to 60% since July 2021.

### Vaccination against COVID-19

**Fig. 5:**
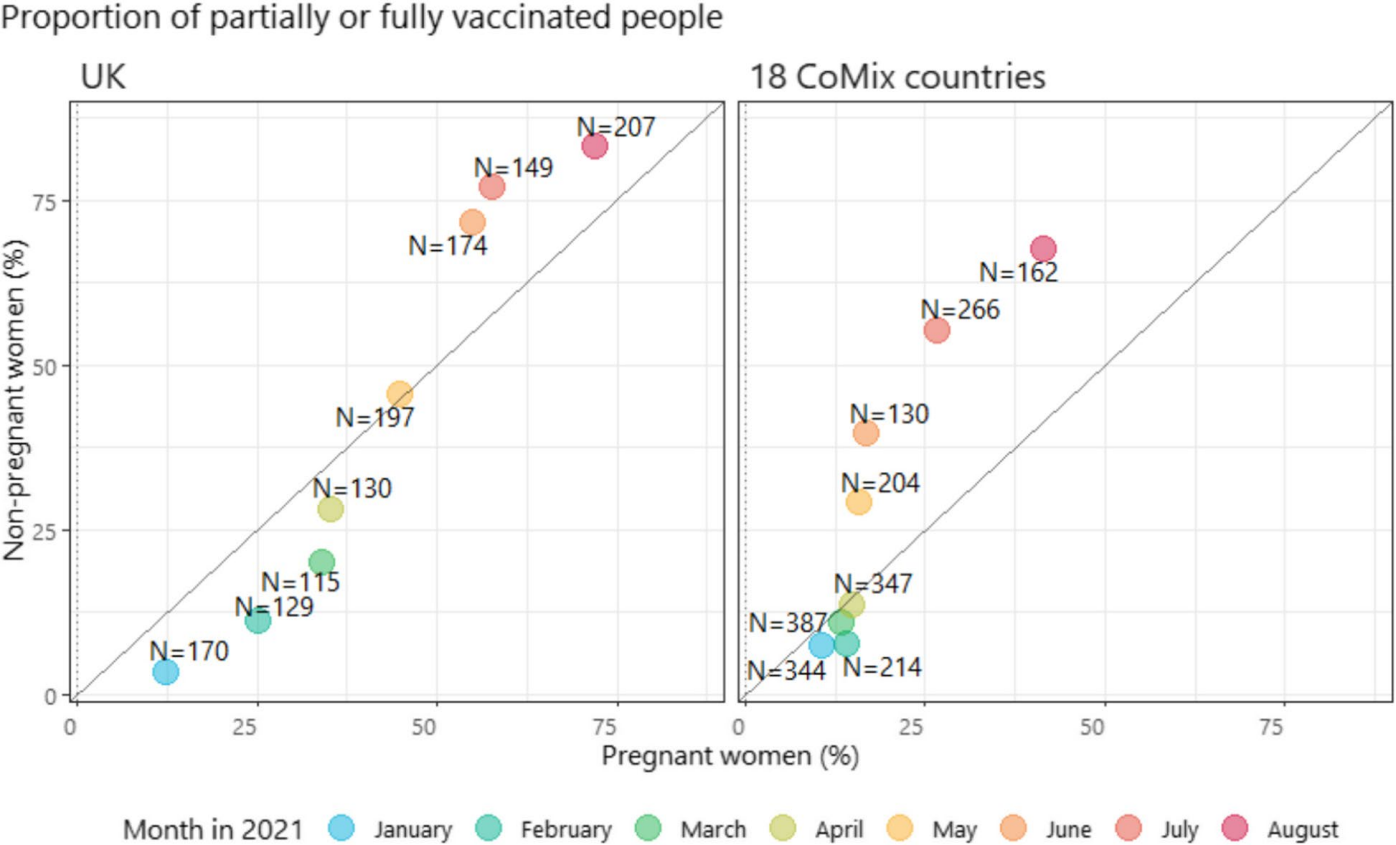
Monthly proportion of pregnant and non-pregnant women who report having been fully or partially vaccinated against COVID-19 in 18 European countries and in the UK. Note: The numbers on the graph indicate the numbers of surveys completed by pregnant women in that month

We plotted the monthly vaccination coverage among pregnant women against that of non-pregnant women (Fig. 5). Between January and March 2021, vaccine coverage was < 10% in both groups of women in 18 European countries (although only 7 countries contributed data to this period). Since May 2021, vaccination coverage in non-pregnant women began to increase whilst coverage in pregnant women remained broadly static. In June, for instance 55% of non-pregnant women in European countries were partially or fully vaccinated. This contrasted with 25% of pregnant women being partially or fully vaccinated. A similar pattern was also observed among UK participants (Fig. 5).

## Discussion

We conducted a large, longitudinal survey that quantifies the changes in social contacts, perception and vaccination over the first 18 months of the COVID-19 pandemic. This period encapsulates time periods of varying strictness of NPIs – ranging from highly restrictive “lockdown” (e.g., second quarter in 2020) to close to no restrictions at all (summer 2021). Mean contact rates were lower in pregnant women compared to non-pregnant individuals throughout the study period, driven by fewer contacts made at work. In social settings outside of participants’ home, however, pregnant women made more contacts under all NPI levels; and maintained a similar levels of contacts in “non-essential” social settings – such as other people’s home, a place of entertainment or sports – regardless of NPIs. A consistently greater proportion of pregnant women reported to be in isolation or quarantine due to COVID-19 and were concerned that they would have serious COVID-19 throughout the study period. The use of face-covering was also more common in pregnant women in the earlier stage of the pandemic. Since June 2021, vaccination coverage in pregnant women fell short of that in non-pregnant people in all study countries.

Our main findings on more isolation/quarantine related to COVID-19 among pregnant women (might be in part due to higher percentage of pregnant women in households with children, whom might have higher exposure in childcare settings or school), and fewer social contacts than non-pregnant individuals are in line with those found in previous studies. During the pandemic, pregnant women had been demonstrated to meet their social support needs mostly through virtual means, with only approximately one in five meeting with someone in-person [25, 26]. Our understanding of how digital communication technology to attenuate people’s sense of isolation and loneliness remains, however, limited [27]. Our study revealed more contacts in non-essential social settings among pregnant women, suggesting a minimal level of social contacts that women reporting as being pregnant may consider as vital. Connections and support from people in one’s network may be viewed particularly important for women entering parenthood [28, 29]. As the pandemic evolves and continues, additional attention by healthcare workers and dedicated programs can help care for maternal social need, especially from in-person interactions in settings of high rates of COVID-19 infection and strict NPIs. While the current study focused on the women reporting as being pregnant, continued support in the postpartum period is also essential for ongoing maternal, child and family health.

Pregnant and reproductive-aged women account for a large proportion of the population with particular concerns regarding vaccination against COVID-19. Studies conducted in UK, Ireland, and the United States revealed greater vaccine hesitancy in pregnant women compared to non-pregnant women of the same age [30, 31]. This pattern is reflected in our findings (apart from the first few months of the vaccination programme when healthcare workers were among the prioritized groups to receive the vaccine). We combined data on vaccine coverage in 18 different European countries – due to low number of participants from this vulnerable group in each individual country – bearing in mind the different COVID-19 maternal immunization policies across countries, and over time in a country. The finding on lower vaccine coverage in pregnant women than in non-pregnant women in this study is likely due to a mixture of policies barring access to vaccines based on pregnancy status and low uptake by pregnant women where the vaccine is offered. Evolving recommendations and misinformation had led to some understandable hesitancy among pregnant women when COVID-19 vaccines were first authorized. Since the end of our data collection, evidence about the safety and effectiveness of the COVID-19 vaccines in pregnancy has become more available, providing assurance that the benefits of vaccination (especially in settings with high rates of COVID-19 infection) is in favour of vaccination for pregnant women [32, 33]. Those responsible for maternal and neonatal health in many countries have now approved the COVID-19 immunization in pregnancy [34]. In a recent Scottish study, about one in three women giving birth in October 2021 had two doses of vaccine, compared to > 75% in general women population of 18–44 years. Vaccine coverage may remain considerably lower in pregnant women. Strategies to address low vaccine uptake in pregnant women is imperative to protect the health of women and babies in the ongoing pandemic [35].

The findings presented in this study should be interpreted with several limitations in mind. First, more than half of our data came from the UK which may have obscured patterns from other countries. Relatedly, for the analysis of COVID-19 vaccination in countries other than the UK, we grouped 18 countries together due to data availability. This approach neglected variations in vaccination schedules and the evolution of vaccine policies on pregnancy in individual countries. Both risk perceptions to COVID-19 and vaccine hesitancy also likely differ across countries and change over time. Future research is highly warranted as more data become available. Second, we did not collect data on gravidity and gestational age from pregnant women, which likely have important relationships with perceived need of social support and vaccine acceptance. Factors associated with subgroups of pregnant women requiring particular attention should be thoroughly explored in future studies. We were also unable to confirm self-reported pregnancy status or any changes in pregnancy outcome, a transitory status. This can impact our analysis since difference pregnant statuses can influence social contact behaviour. Third, we found a higher proportion of pregnant women who were healthcare workers than non-pregnant individuals; and those who reported as being pregnant had fewer work contacts. Our study sample might present some selection bias, or have captured a phenomenon wherein pregnant healthcare workers worked remotely, or were redirected to roles with less exposure to people. Forth, pregnant and non-pregnant individuals may have interpreted the questions on isolation and quarantine due to COVID-19 differently. Terminologies such as “isolation”, “quarantine” and “social distancing” are distinctively different yet somewhat similar notions that might be misinterpreted, especially for high-risk people who have been given stricter social-distancing guidelines. Our finding on isolation and quarantine might have over-estimated the proportion of pregnant women who were truly in isolation or quarantine. Fifth, the study was conducted online using a quota-based sample of individuals who had agreed to participate in a marketing survey. The recruitment method may not lead to a representative sample of pregnant women, and is particularly prone to bias towards people with access to the internet and who may be reached by online advertising campaigns. The other limitations of our online survey have been documented elsewhere [15, 19].

## Conclusion

The COVID-19 pandemic quickly transformed the global social landscape as widespread NPIs reshaped social behaviour, especially for the most vulnerable. The vigilance with social behavioural guidelines given to pregnant women has limited their access to seek in-person support from others during pregnancy – typically a time of heightened social need. Recognition of such a need and its impact on intrapartum wellbeing remain critical in the ongoing COVID-19 pandemic, as well as in future large-scale infectious disease outbreaks.

The lack of relevant data on COVID-19 vaccine safety during pregnancy at the beginning of vaccines authorization left authorizing bodies and pregnant women uncertain about vaccinating against COVID-19. As the generation of further evidence on vaccine safety during pregnancy continue, effective strategies to disseminate evidence can provide reassurance and facilitate informed pregnancy vaccine decisions in this vulnerable group.

## Supplementary Information

**Additional file 1:** **Supplementary Figure S1.** Oxford COVID-19 Government Response Tracker Stringency Index in 21 study countries. **Supplementary Figure S2.** Covid-19 vaccine policies on pregnancy in 18 CoMix countries between March and September 2021. **Supplementary Table S1.** Number of participants and number of completed surveys by country.

## Data Availability

The datasets generated and analysed the current study is available from http://www.socialcontactdata.org/data/.
